# Draft Genome Assembly of a Filamentous Euendolithic (True Boring) Cyanobacterium, *Mastigocoleus testarum* Strain BC008

**DOI:** 10.1128/genomeA.01574-15

**Published:** 2016-01-28

**Authors:** Brandon S. Guida, Ferran Garcia-Pichel

**Affiliations:** Arizona State University, School of Life Sciences, Tempe, Arizona, USA

## Abstract

*Mastigocoleus testarum* strain BC008 is a model organism used to study marine photoautotrophic carbonate dissolution. It is a multicellular, filamentous, diazotrophic, euendolithic cyanobacterium ubiquitously found in marine benthic environments. We present an accurate draft genome assembly of 172 contigs spanning 12,700,239 bp with 9,131 annotated genes with an average G+C% of 37.3.

## GENOME ANNOUNCEMENT

Some microorganisms, known as euendoliths, have the remarkable capacity to excavate and grow into solid mineral phases, most typically carbonate minerals, often in the face of imposing physiological hurdles (1, 2). Cyanobacterial euendoliths are ubiquitous constituents of exposed carbonate outcrops, terrestrial and aquatic. In the marine environment they can represent important primary producers in carbonate-dominated systems (3). Euendolithic cyanobacteria play several significant roles in the dissolution of coral skeletons (4), and the erosion of coastal and terrestrial carbonate outcrops (5, 6). Their activity also impacts the viability of natural and farmed bivalve populations (7). Despite their ecological importance, currently no accurate genomic information is available for any euendolith.

*Mastigocoleus testarum* is a filamentous, true-branching, diazotrophic, morphologically and developmentally complex, cyanobacterial species, described as a euendolith in 1886 (8), an important pioneer of endolithic communities (9), and a pest for bivalve fisheries (10). A type strain, BC008, isolated from a shell fragment (11), represents the only working physiological model organism for the study of cyanobacterial euendoliths (1). While we have gained important insights into the mechanistic underpinning of carbonate dissolution using BC008, we are hampered by a lack of genetic information, which would enable comparative genomics, transcriptomics, and perhaps even the development of a genetic manipulation system and advances in its management in aquaculture.

*M. testarum* strain BC008 was grown as a unicyanobacterial, but not axenic, culture, in PES-30 medium (12) under a 16/8 h light/dark diel cycle at room temperature. Genomic DNA was isolated as previously described (13) and two separate Illumina MiSEQ paired-end (PE) libraries were created. One, 2 × 150 (lib1), was generated by the Joint Genome Institute and another, 2 × 300 (lib2), by the Translational Genomics Institute. The libraries revealed the presence of three distinct, divergent 16S rRNA sequences. In addition to the dominant 16S ribosomal sequence for *M. testarum*, the cultures contained 2 contaminants, one in the *Rhizobiales* and one related to *Hyphomonas* (99.7% identity). The genomic data set was thus treated as a metagenome. After quality filtering, both libraries combined contained over 7.2 Gbp of sequence. Each library underwent independent assembly using the iMETAMOS (14) pipeline of METAMOS (15). The best assembly was used for each library and the resultant contigs were assembled together using Geneious (16). Because both contaminants had a high G+C content genome (>50%), all contigs above that mark were removed. Contigs longer than 3 kbp were scaffolded using SSPACE (17), resulting in a final draft assembly of 172 scaffolds (>5 kbp, *N*_50_:145,830; mean length: 65,170) representing 12,700,239 bp of genomic DNA with an average G+C% of 37.3, which is among the largest bacterial genomes reported. Gene prediction and annotation was done using the NCBI prokaryotic genome annotation pipeline (18) resulting in 9,131 genes, 73 tRNA loci, and 6 rRNA loci (two complete rRNA operons). Secondary metabolite gene cluster prediction was done using the antiSMASH server (19) and resulted in 22 predicted gene clusters indicating the potential biosynthesis of shinorine, nostopeptolide, hectochlorin, and staurosporine, among others.

### Nucleotide sequence accession numbers.

This whole-genome shotgun project has been deposited at DDBJ/EMBL/GenBank under the accession number LMTZ00000000. The version described in this paper is version LMTZ01000000.
